# The British Journal of Psychiatry affirms its support of drug and alcohol research

**DOI:** 10.1192/bjp.2025.10501

**Published:** 2025-11-28

**Authors:** Emmert Roberts, Emily Finch, Anne Lingford-Hughes

**Affiliations:** National Addiction Centre, Institute of Psychiatry, Psychology and Neuroscience, King’s College London and the South London and the Maudsley NHS Foundation Trust; Chair of the Faculty of Addiction Psychiatry at the Royal College of Psychiatrists and the South London and the Maudsley NHS Foundation Trust; Chair of the Addiction Healthcare Goals, Office for Life Sciences, Imperial College London and the Central and North West London NHS Foundation Trust

## Abstract

Internationally, drug and alcohol-related morbidity is at its highest recorded levels, yet these modifiable risk factors often remain overlooked within systems of healthcare delivery and research. We seek to reaffirm the ongoing commitment of *BJPsych* to publishing and promoting drug and alcohol research and encourage the submission of high-quality articles.

## Editorial

International research consistently demonstrates that alcohol and tobacco use routinely rank at the top of modifiable risk factor league tables as contributing to global morbidity and mortality, with illicit drug use also often a feature of top ten lists. (1) Despite alcohol and drug-related pathology affecting almost every organ system, and being frequent features of presentations to medical, surgical and psychiatric specialities, there is often a perception that management of an individual’s underlying drug or alcohol use is not the purview of the current treating professional. (2) This situation appears unfortunately paralleled within many areas of healthcare research, with typically low numbers of studies recruiting participants with co-morbid drug and alcohol problems.

Alcohol, tobacco and/or other drug use may have material and lasting impacts on both individual and population-level physical and mental health. As academic journals remain trusted sources of evidence and research, provisioning a wide array of clinicians, researchers and policymakers, this article argues that encouragement, support and publication of research in this area is vital and may have a substantial and positive impact on reducing the record levels of preventable substance-related harm currently manifest across the globe. (1, 3, 4) The *BJPsych* editorial board acknowledges the clinical importance of these issues, recognises that more needs to be done, and seeks to reaffirm the *BJPsych’s* commitment to publish drug- and alcohol-related research. This editorial serves as a prompt and a reminder to all that the *BJPsych* is keen to publish high quality research in this field.

## Why is the *British* Journal of Psychiatry currently acutely aware of these issues?

Over the past decade the United Kingdom has experienced record levels of drug- and alcohol-related harm with simultaneously observed declines in the number of people accessing drug- and alcohol treatment services and in the number of researchers active in this area. (5) In 2024 the *Drug and Alcohol Treatment and Recovery Workforce Census* in England reported that fewer than 0.2% (n=59) of staff were medically trained specialists, the number of whole time equivalent Consultant posts having more than halved over the previous decade. (6) Contemporaneous legislative changes also resulted in substantial reductions in funding and changes to the commissioning structure of community drug and alcohol services, with most English service contracts now subject to competitive tendering. Notably, over two thirds of services are now provided by the voluntary, community and social enterprise (VCSE) sector as opposed to the National Health Service (NHS). (5) This has led to a significant reduction in the ability to train registered professionals and in the availability and integration of the drug and alcohol workforce within healthcare settings during a period over which the number of national drug- and alcohol-related deaths has continued to set tragic new records with each passing year. (3, 4) Given the ubiquity of ill-health attributable to drug and alcohol use, as evidenced by the staggering statistic that over a third of patients entering UK hospitals are estimated to harmfully use alcohol, (7) models of both healthcare delivery and research that artificially separate and silo the treatment and understanding of problematic drug and alcohol use inevitably result in systems that are inefficient and hinder the provision of holistic person-centred care.

Given the currently observed extreme levels of drug- and alcohol-related harm one might assume that the orientation of healthcare and research ecosystems may shift in response. However, ongoing stigma, and the persistent commercial framing of problematic drug and alcohol use as an individual moral failure, continue to contribute to these modifiable risk factors being afforded Cinderella status. (2) The recent Independent Review of Drugs noted the parlous state of the national drug and alcohol treatment system following over a decade of sustained funding cuts, with the review’s recommendations paving the way for renewed investment in both treatment delivery and research infrastructure. (5) Multiple organisations have subsequently led initiatives seeking to specifically improve integration between drug and alcohol and wider mental health provision with a focus on the management of individuals with Co-occurring Substance Use and Mental health disorders (Co-SUM). (8) Given the high number of individuals presenting with these disorders, it is vital that funding agencies support calls that acknowledge this comorbidity and that the content of *BJPsych* and other non-specialist mental health journals reflects the clinical realities faced by a general psychiatric audience. We would hope that authors of any future research outputs, including those that may be generated via this renewed energy and funding, will now consider *BJPsych* a receptive and suitable home.

## A commitment to champion and publish relevant research

The *BJPsych* recently published an editorial pledging its commitment to publish science that is conducted with integrity and rigour, and equally importantly that which concerns the provision of care to vulnerable populations. (9) In the current international landscape, changes to research priorities and funding structures that may restrict the study of marginalised communities who may have experienced trauma and/or interpersonal violence are likely to disproportionally, and indeed negatively, affect individuals who use drugs and alcohol.

The ultimate goal of professions working across public health and the psychological, psychiatric and neuroscientific landscape is to alleviate the distress and suffering caused and conferred by mental and behavioural disorders. The harms related to alcohol and drug use should not be subject to assumptions, such as the oft quoted assertion, that they fall outside the remit of the sphere of influence of these professions, or that they are somehow different or less important than other modifiable risk factors. We are acutely aware as an editorial board that, when discussing drug and alcohol use, *BJPsych* affiliates often anecdotally report hearing colleagues state that the journal “*doesn’t publish that kind of research*” or “*they only care about cannabis*”. Whilst it would be presumptuous of *BJPsych* to think we might be able to reorientate the field entirely, what is perhaps in our power is to recommit to publish research that aims to improve the lives of the millions of individuals who experience harm following use of drugs and alcohol. Put another way, we are renewing our vows, and we encourage editors of non-specialist journals worldwide to join us and do the same so that we can jointly ensure that the modifiable risk factors causing the highest levels of morbidity and mortality globally are tackled with the urgency they merit.

## References

[1] Degenhardt L, Charlson F, Ferrari A, Santomauro D, Erskine H, Mantilla-Herrara A (2018). The global burden of disease attributable to alcohol and drug use in 195 countries and territories, 1990–2016: a systematic analysis for the Global Burden of Disease Study 2016. The Lancet Psychiatry.

[2] Van Boekel LC, Brouwers EP, Van Weeghel J, Garretsen HF (2013). Stigma among health professionals towards patients with substance use disorders and its consequences for healthcare delivery: systematic review. Drug and alcohol dependence.

[3] Alcohol-specific deaths in the UK Statistical bulletins.

[4] Deaths related to drug poisoning in England and Wales Statistical bulletins.

[5] (2021). Independent review of drugs by Professor Dame Carol Black.

[6] Drug and Alcohol Treatment and Recovery Workforce Census.

[7] Roberts E, Morse R, Epstein S, Hotopf M, Leon D, Drummond C (2019). The prevalence of wholly attributable alcohol conditions in the United Kingdom hospital system: a systematic review, meta-analysis and meta-regression. Addiction.

[8] Co-occurring substance use and mental health disorders (CoSUM).

[9] Malhi GS, Adlington K, Al-Diwani A, Ali S, Arya R, Baldwin DS (2025). The value of mental science: we publish what matters. The British Journal of Psychiatry.

